# Hydroavacciniforme on a dark skin with mucosal involvement

**DOI:** 10.11604/pamj.2016.23.71.9199

**Published:** 2016-03-09

**Authors:** Kelati Awatef, Mernissi Fatima Zahra

**Affiliations:** 1 Hospital University Hassan II, Department of Dermatology, Faculty of Medicine, Fez, Morocco

**Keywords:** Hydroa vacciniforme, dark skin, mucosal involvement

## Image in medicine

Hydroa vacciniforme is a rare idiopathic photodermatosis uncommon in dark skinned patients, which usually begins in childhood before the age of 10 years old and disappear in adolescence, it begins symmetrically in photo exposed areas as a sensation of skin burning in less than 24 hours of sun exposition, and then a vesicular rash develops, become umbilicated and secondarily crusted. Within weeks, the scars heal and leave a residual varioliform scar appearance, the condition generally improves with regular use of high protection sunscreens and resolves during adolescence. In patients who do not respond to conservative treatment, use of systemic agents has been reported like psoralen with exposure to ultraviolet A [PUVA], ultraviolet B TL-01phototherapy, antimalarial agents and immunosuppressive medication. Our patient is a 10-year-old child -without pathological antecedents- who consulted for atrophic depressed scars after a vesicular rash, which begin with a sensation of skin burning a day after intense sun exposure. These scars are covered by hemorrhagic crusts in some areas and appear on exposed areas: face, ear, hands and forearms with erosive lesions of the lower lip. The Child reports that this is the second episode with an interval of 6 months between the 2 episodes.

**Figure 1:**
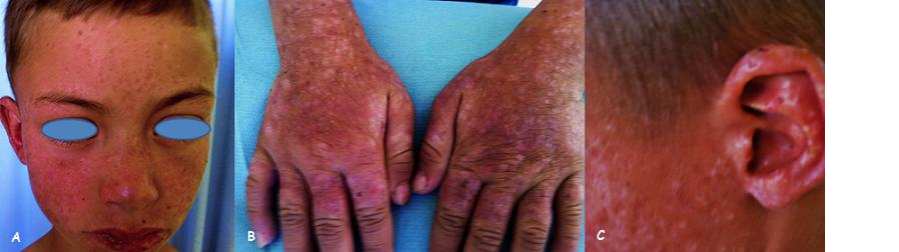
A) varioliform scars covered by hemorrhagic crusts on the face, with erosive lesions of the lower lip; B) hypopigmented varioliform scars on the dorsal area of the hands and forearms; C) hypopigmented varioliform scars on the pavilion of the ears and the helix

